# Prospective controlled study to compare the effects of a basic patient safety course on healthcare worker patient safety culture

**DOI:** 10.1186/cc14594

**Published:** 2015-03-16

**Authors:** L Ling, G Joynt, A Lee, W Samy, H Fung, CD Gomersall

**Affiliations:** 1 The Chinese University of Hong Kong, Shatin, Hong Kong

## Introduction

It is estimated that about one in 10 patients may be harmed by adverse events during their hospital stay [1]. Transforming organizational culture to improve patient safety culture is considered important. We conducted a prospective, controlled study to assess the impact of a standardized patient safety course on an ICU's patient safety culture, using a validated patient safety culture assessment tool.

## Methods

Staff from two ICUs - ICU1 (tertiary referral hospital) and ICU2 (district hospital) - in Hong Kong were recruited to compare changes in the measured safety culture before and after a patient safety course. The BASIC Patient Safety course was only administered to staff from ICU1, and safety culture was assessed in both units before and after, using a survey based on the Hospital Survey on Patient Safety Culture [2]. Relative risk (95% CI) of improvement: baseline to follow-up in hospitals in patient safety domains, adjusted for duration of work in the unit (≤10 years vs. >10 years), was calculated. Responses were coded according to the Survey User's Guide, and positive response percentages for each patient safety domain were compared with the 2012 Agency for Healthcare Research and Quality (AHRQ) ICU sample of 36,120 respondents.

## Results

Preintervention and postintervention period response rates for ICU1 were 88.1% (37/42) and 79.3% (23/29); and for ICU2 63% (20/32) and 63% (15/24). Post intervention, compared with ICU2, ICU1 showed significantly improved perceptions of teamwork within the hospital unit, RR (95% CI for difference between ICUs) 1.55 (1.10 to 2.19, *P *= 0.01); and overall perception of safety, 1.94 (1.11 to 3.37, *P *= 0.02); but not increased frequency of reporting mistakes, 0.90 (0.33 to 2.49, *P *= 0.84). Overall, ICU1 demonstrated a greater improvement in positive responses in five safety culture domains than staff from ICU2. Patient safety culture indices were generally poorer in the two ICUs than the average ICU in the AHRQ database.

## Conclusion

The study provides supportive evidence that a structured, reproducible short course on patient safety is associated with a general improvement in the ICU's patient safety culture, measured with a validated safety culture assessment tool.
